# DESI–MS as a tool for direct lipid analysis in cultured cells

**DOI:** 10.1007/s10616-014-9734-z

**Published:** 2014-05-07

**Authors:** Anna Bodzon-Kulakowska, Tomasz Cichon, Agnieszka Golec, Anna Drabik, Joanna Ner, Piotr Suder

**Affiliations:** AGH University of Science and Technology, Kraków, Poland

**Keywords:** Desorption electrospray ionization (DESI), Cell culture, Lipid analysis, Oxidative stress

## Abstract

Desorption electrospray ionization may be used as a fast and convenient method for analysis and identification of lipids in the cell culture. Oxidative stress, which usually involves changes in lipids, was used as a model of pathology to show the utility of this analysis methodology. This paper addresses the surface preparation of cell culture slides, induction of oxidative stress, and cell monolayer culture preparation as well as optimization of the analysis. Advantages and drawbacks of the method were also discussed.

## Introduction

**Fig. 1:**
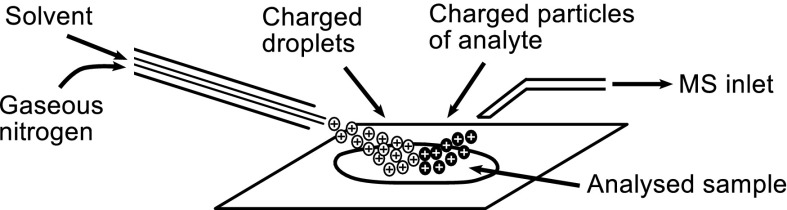
DESI ion source

Desorption electrospray ionization (DESI) is a mass spectrometry imaging (MSI) method used in the biological sciences for surface analysis. An electrospray emitter is used to generate charged microdroplets which are directed at the surface for analysis, where they create a thin liquid film which dissolves the analyte(s). Secondary microdroplets containing the analyte are produced by the impact of subsequent primary droplets. The analyte is introduced into the MS inlet where the ions are analysed by mass spectrometer (Girod et al. 2010a; Takáts et al. 2004) (Fig. 1).

Compounds below 2,000 Da (such as lipids) can be measured and their distribution on the surface visualized, while particular molecules may be identified using MS/MS fragmentation. Additional advantages are that DESI does not require laborious sample preparation and can be performed at ambient conditions. Its spatial resolution reaches a pixel size of ca. 50 × 50 μm or better (down to 10 × 10 μm) with modification of the DESI source (Laskin et al. 2012). Taken together, DESI is a fast and convenient tool for lipid analysis in biological samples (Eberlin et al. 2011).

Oxidative stress is a pathological state of the cell/organism where the amount of oxygen radicals increases but cannot be counterbalanced by the internal antioxidant defense system, or when the level of oxidative radicals remains at a physiological state, but the defense system is impaired. This may lead to the oxidative damage of proteins, lipids, and DNA, which usually leads to apoptosis (Feeney et al. 2008). Lipids are especially sensitive to this pathophysiology and undergo peroxidation, which results in a number of highly-reactive species that can modify other proteins and lipids, yielding aldehydes and polymerized carbonyl compounds (Weismann and Binder 2012). As lipid alterations are involved in oxidative stress, the model in this study was chosen to show the usefulness of DESI lipid analysis in cell culture.

## Materials and methods

If not otherwise stated, all chemicals were purchased from Sigma-Aldrich (Poznan, Poland) and were of the highest purity available.

### Surface preparation for cell culture

**Fig. 2:**
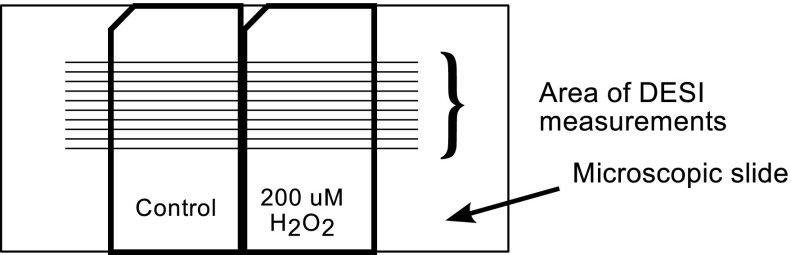
DESI measurements—arrangement of the glasses with the monolayer cell culture on the DESI holder

The DESI ion source (OMNIspray, Prosolia, Indianapolis, IN, USA) was equipped with a holder for standard microscopic glass slides (75 × 25 mm) as a base for surface scanning. To combine cell monolayers from various experiments on a single holder, cells were cultured on glass slides (15 × 25 mm) and individual glasses were combined on a DESI holder (Fig. 2). The upper-left corner of each glass was cut at the angle of 45° to allow for immediate identification of the upper surface of the glass slide.

Before cell culturing, glass slides were washed with hexane for 5 min in an ultrasonic bath (Sonorex-Digitec, Bandelin-Electronic, Berlin, Germany) and then with 95 % ethanol for 5 min. The remaining work was done in a laminar airflow cabinet (NU-480, NuAire Inc., Plymouth, MN, USA) to ensure aseptic conditions. The ethanol was evaporated, slides washed twice with sterile water and covered with a 0.02 mg/ml solution of poly-l-lysine (30–70 kDa; P2636, Sigma-Aldrich) overnight. Immediately before cell culturing, the slides were removed from the poly-l-lysine and washed twice with PBS.

### Cell culture

A fibroblast cell line (Balb/3T3 cells, Ludwik Hirszfeld Institute of Immunology and Experimental Therapy, Polish Academy of Sciences, Wroclaw, Poland, stored in a liquid nitrogen) was thawed at 37 °C. The cell suspension was centrifuged at 300×*g* for 7 min, the cryoprotectant medium removed and the cells were resuspended in Eagle’s Minimal Essential Medium (M2279, Sigma-Aldrich) supplemented with 10 % v/v Foetal Bovine Serum (F9665, Sigma-Aldrich), 1 % v/v antibiotic antimycotic solution (A5955, Sigma-Aldrich) and 1 mM glutamine (49419, Sigma-Aldrich). The cells were centrifuged at 300×*g* for 4 min, resuspended in cell culture medium, seeded on the poly-l-lysine coated slides and cultured at 5 % CO_2_, 37 °C and 95 % relative humidity (DH5810E, NuAire Inc.) for 6 days until confluent.

### Oxidative stress

The simplest method to induce oxidative stress in cell culture was to disturb the prooxidant-antioxidant balance by increasing radical load, which can be accomplished by adding hydrogen peroxide (or other agents) to the cell culture medium (Gille and Joenje 2002). Glass slides with confluent cell monolayers were removed from the Petri dish and placed in a new container with a new portion of medium (control) or medium supplemented with H_2_O_2_ (200 μM) for 1 h. One set of glass slides was used for DESI analysis and the other for analysis of cell viability by trypan blue staining (Patterson 1979).

### Cell culture preparation for DESI analysis

Immediately before DESI, the medium was removed from the Petri dish containing the glass slide with the cell monolayer. To remove salts and other remainings of the cell culture medium, the slide was rinsed twice with a volume of warm (37 °C) 150 mM ammonium acetate buffer, pH 7.1 (A7330, Sigma-Aldrich) for 5 s. The glass slide was removed from the dish, dried using a stream of dry nitrogen directed at the surface of the cell monolayer and frozen at −80 °C until DESI analysis. The isotonic ammonium acetate solution was volatile enough to evaporate quickly (Piwowar et al. 2013).

### DESI analysis

Glass slides with control and hydrogen peroxide-treated cell monolayers were placed into the DESI holder (Fig. 2). During the imaging experiments, cell monolayers were scanned using a 2D moving stage in horizontal rows separated by a 0.2 mm distance, and 50 rows were measured at 100 μm/s with a single mass spectrum saved every 1.5 s (spatial resolution of ca. 170 dpi). A methanol : water solution (1:1 v/v) containing 1 μM surfactin was sprayed at a constant flow rate of 2.0 μl/min. The mixture of water and methanol is a standard solution used for DESI analyses and the addition of surfactin enhanced signal quality, especially in the negative ion mode. Control and 200 μM H_2_O_2_-treated cells were measured during a single analysis (Fig. 2), and Data Analysis ver 4.0 software (Bruker-Daltonics, Bremen, Germany) was used for spectral analysis, while the BioMap freeware (http://www.maldi-msi.org) (Novartis, Basel, Switzerland) was used for image generation.

An DESI OMNIspray ion source combined with an AmaZon ETD MS (Bruker-Daltonics) was operated under the HyStar ver. 3.2 software supervision (Bruker Daltonics). HyStar coordinated work of the Omnispray 2D software (Prosolia) controlling the DESI stage movements, and the Bruker’s TrapControl ver. 7.0 software (Bruker Daltonics) controlling mass spectrometer activity. Mass spectrometer settings were as follows: scan range 300–950 *m*/*z*, capillary voltage of +4,200 V, ion trap operating in the ICC mode set to 300,000 ions/cycle, maximal ion accumulation time of 200 ms and a heated capillary temperature of 220 °C. The negative ion mode was selected, as it yielded more information during lipid analysis than positive polarization. The most abundant peaks from the cell culture sample were further identified using MS/MS fragmentation (collision induced dissociation energy 1.2 V, precursor isolation width 4.0 Da).

### Statistics

Eighty scans were taken to obtain accumulated spectra for every single line from the analysed samples (control and 200 μM H_2_O_2_). The intensities of each chosen peak were averaged, and the standard deviations and *p* values were calculated using the Student’s *t* test.

## Results

### Oxidative stress and cell viability

After 1 h of incubation in the appropriate media, a set of glass slides was subjected to viability test using trypan blue staining. In the control sample and cells subjected to oxidative stress, the viability of the cells was unchanged. However, cells subjected to the 200 μM of H_2_O_2_ started to show morphological signs of oxidative stress by changing their irregular flattened, extended shape and rounding (Kiyoshima et al. 2012).

### DESI analysis

**Fig. 3:**
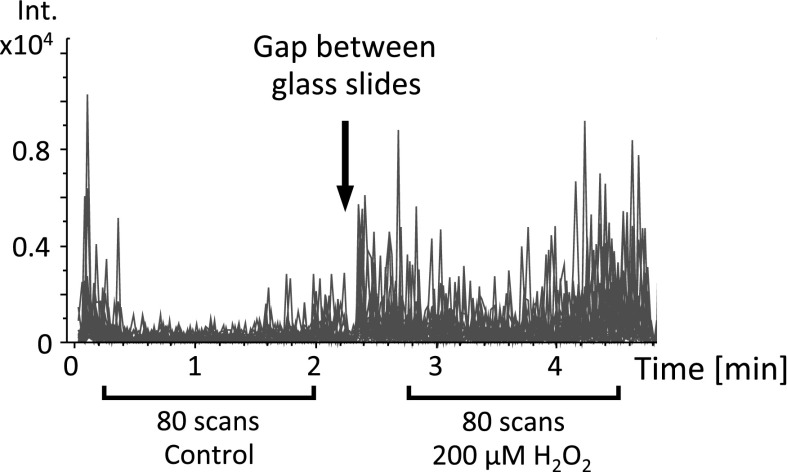
Selection of the mass spectra (scans) for averaging. The plotted *lines* represent a chromatogram of extracted ion at the 885.5 *m/z* peak, characteristic for the areas covered by cells. *Arrow* shows the gap between the glass slides with control cells and cells treated with 200 μM H_2_O_2_

To obtain average spectra for each sample (control and 200 μM H_2_O_2_), 80 mass spectra were accumulated for every surface (Figs. 2 and 3). From the accumulated spectra, ions of interest were selected, and the peaks corresponding to particular lipids, as well as those expected to originate from the background, were considered.

**Fig. 4:**
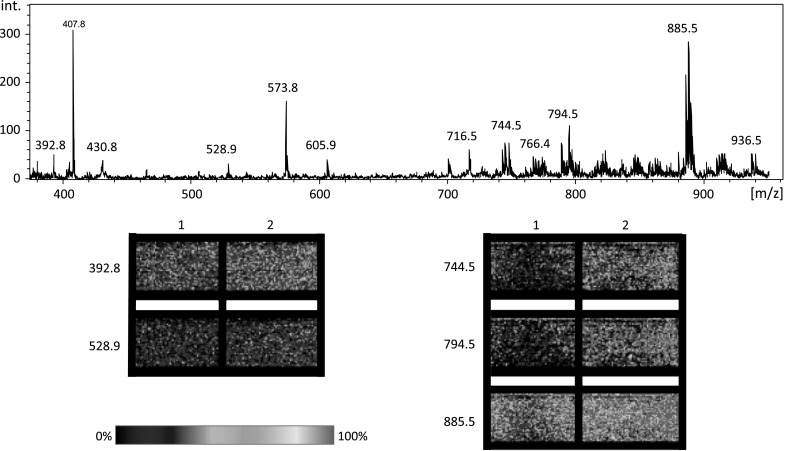
Average spectrum from the cell culture treated with 200 μM of hydrogen peroxide. Below the spectrum are images from Biomap indicating the abundances of the selected peaks in every sample (*1* control, *2* 200 μM H_2_O_2_)

Figure 4 shows the spectrum from cells treated with 200 μM of hydrogen peroxide, averaged from the 80 spectra. The peaks corresponding to particular lipids were selected for analysis, as well as some peaks from the background (for the comparison purpose—as a negative control).

**Fig. 5:**
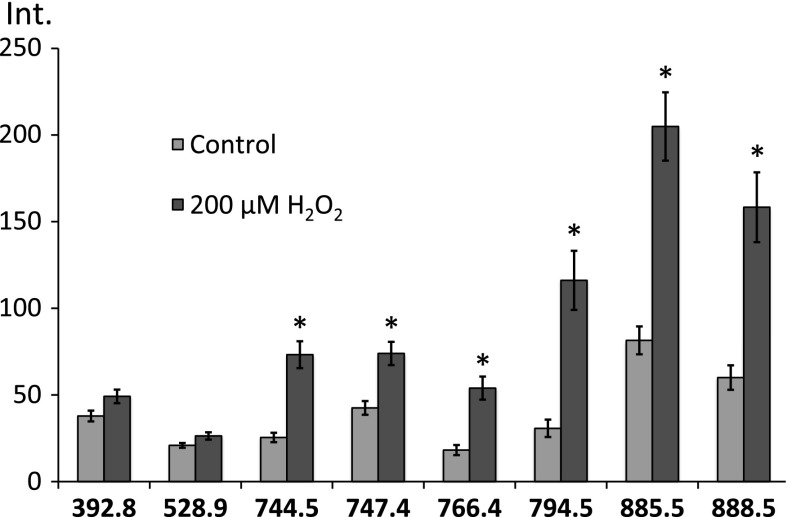
Fluctuation of selected ion intensities after treatment with hydrogen peroxide. Peaks at 392.8 and 528.9 were background, and indicated insignificant variability among samples, in contrast to those representing lipids, shown on the graph. Differences between corresponding values, for the same peaks, marked with an *asterisk* are significant (*p* value <0.05)

Changes in peak intensities after hydrogen peroxide administration are shown in Fig. 5.

### MS/MS fragmentation

DESI allowed identification of analysed compounds using MS/MS fragmentation. In the negative ion mode, identification of lipids is not a very demanding task. Usually these compounds form pseudomolecular ions [M−H]^−^, which fragment by losing a characteristic portion attached to the sn-3 position of the glycerol backbone (Table 1). Characteristic peaks from such fragmentation may be present on the spectrum.

**Table 1:** Characteristic group loss from sn-3 position for lipids easily detected during MS/MS analyses

Lipid name	Abbreviation	Group loss from sn-3 position
Glycerophosphocholine	GPCho (PC)	[M−H−15]^−^, [M−H−60]^−^, [M−H−84]^−^
Glycerophosphoethanolamine	GPEtn (PE)	[M−H−104]^−^
Glycerophosphoglycerol	GPGro (PG)	[M−H−74]^−^
Glycerophosphoinositol	GPIno (PI)	[M−H−162]^−^
Glycerophosphoserine	GPSer (PS)	[M−H−87]^−^
Plasmanylphospholipid	Plasm-PE	[M−H−43]^−^
Sulfatide	ST	[M−H−18]^−^

The MS/MS spectra of lipids showed losses of two different neutrals, carboxylic acid [M−H−R_1,2_CH_2_COOH] and the corresponding ketene [M−H−R_1,2_CH=C=O]^−^. Examples of masses for pseudomolecular ions and neutrals characteristic for different fatty acyl substituents are listed in the Table 2. Lipids were identified according to Tables 1 and 2 in addition to data from the literature (Manicke et al. 2008; Hsu and Turk 2009, 2005).

**Table 2:** Masses for pseudomolecular ions and neutrals characteristic for different fatty acyl substituents

	Carboxylic acid [M−H]^−^	Carboxylic acid	Ketene
(14:0)	227	228	210
(16:1)	253	254	236
(16:0)	255	256	238
(18:2)	279	280	262
(18:1)	281	282	264
(18:0)	283	284	266
(20:4)	303	304	286
(20:3)	305	306	288
(20:2)	307	308	290
(22:6)	327	328	310
(24:1)	365	366	348

**Fig. 6:**
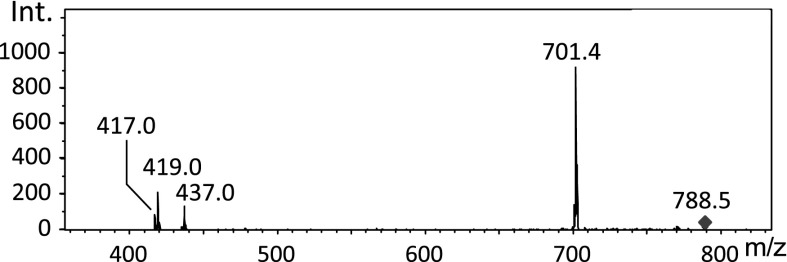
MS/MS fragmentation of the ion at 788.5 *m*/*z*. See the text for details

Moreover, neutral loss for the sn-2 position was more favourable, giving rise to more intense peaks, and the position of the two carboxylic acids may be distinguished. Excellent reviews on this topic may be found in Hsu and Turk (2009) and Girod et al. (2010b). An example of MS/MS spectrum for the ion at 788.5 *m*/*z* is shown in Fig. 6. The main peak on the MS/MS spectrum at 701.4 *m*/*z* resulted from serine loss (788 − 701 = 87), the peaks at 419.0 and 437.0 resulted from neutral loss of 18:1 as a carboxylic acid (788.5 – 87 − 419 = 282.5) and ketene (788.5 − 87 − 437 = 264.5) as well as a small peak at 417 *m*/*z*, which was from neutral loss of 18:0 (788.5 − 87 − 417 = 284.5) (see Table 2). These data indicated the ion at 788.5 *m*/*z* was glycerophosphoserine (18:0/18:1).

Some of the peaks from the cell culture which changed due to oxidative stress were identified (Table 3).

**Table 3:** MS/MS identification of several lipids whose level changed due to oxidative stress

744.5	Glycerophosphoethanolamine PE (18:0/18:1)
747.4	Glycerophosphoglycerol PG (18:0/18:1)
766.4	Glycerophosphoethanolamine PE (18:0/20:4)
885.5	Glycerophosphoinositol PI (18:0/20:4)
888.5	Sulfatide ST (18:1/24:1)

## Discussion

DESI offered a rapid and convenient way of sample measurement/imaging compared to other mass spectrometry imaging techniques based on MALDI and SIMS ion sources that require high vacuum inside the source. The entire procedure for preparing the single glass slide and adherent cell monolayer culture required ca. 30 s with an additional 10 min for drying. DESI construction on the 2D moving stage allowed for placement of glass/plastic slides with adherent cells from various experiments side by side, which allowed the measurements to be performed simultaneously. This was crucial for comparative research between cells treated with various substances or cells with different pathologies.

As with most analytical techniques, DESI was not without drawbacks. The main disadvantage was the limited range of *m/z* that could be analysed. Compounds below 2,000 Da were easily analysed, but for higher masses, a more sophisticated optimization of the system, as well as prescanning of the surface might be necessary. DESI allowed imaging of the surface, but for cell culture analyses, the resolution of the system did not allow for detailed analyses of a single cell. This limitation was due to two variables: (1) the smallest available spraying surface for acquisition of a single pixel, and (2) the analysis time. The DESI interface was suitable for imaging macroscopic objects, such as brain structures (Eberlin et al. 2011). Recent publications about DESI-related analyses indicate that resolutions better than ca. 2,000 dpi are not currently available at the present stage of ion source development (Laskin et al. 2012).

Analysis time was connected to image resolution, and higher resolution required the system to acquire more scans, which served as points (pixels) of the image. Thus, twofold better resolution required fourfold more time to complete the analysis. Another disadvantage of DESI analysis was related to proper optimization of the DESI source. There were several parameters crucial for obtaining a quality spectrum and small changes in source geometry (nebuliser capillary angle, its distance to the surface and distance to the MS inlet), as well as other settings such as nebulising gas pressure, solvent flow and capillary voltage, may affect the analyses. Optimisation based on artificial, easily-ionisable standards (e.g. rhodamine or bradykinin) may be insufficient for more complex samples such as cells cultured on glass (Bodzon-Kulakowska et al. 2014). Thus, additional sets of samples only subjected to ion source optimisation should be prepared. Another thing is that the analysed surface should be as even as possible, because small changes in the distance between nebuliser capillary and the surface may influence peak intensity.

The effects of oxidants may be easily detected in cell monolayers with the aid of DESI-equipped mass spectrometers. Cell cultures did not require advanced preparation methods or any special type of visualization of the changes occurring between samples prior to the MS analysis. With respect to analysis of cellular membrane lipid in various cell cultures, DESI appeared to be the most rapid, convenient, reliable, and sensitive method of analysis. In direct contrast to staining techniques, radioimmunoassays, lipid derivatization or homogenate analyses, DESI did not introduce any additional substances to the sample, and advanced, multi-step procedures were not necessary. Changes in abundances of ions corresponding to lipids under oxidative stress, could be seen. Additionally, using DESI in combination with MS/MS analysis allowed these species to be identified.

Categorical judgements about the biological significance of the effects observed were not possible based on these preliminary experiments, as this work was devoted to evaluating the application of DESI as a tool to analyse cell changes. However, the effects may be connected to several processes in the cells. With the aid of DESI, a direct destructive effect of hydrogen peroxide on the cell membranes of the living cells could be observed. Interactions between reactive oxygen species and the cell membrane could cause partial destruction which would expose lipids, rendering them more susceptible to ionization. This may be supported by the presence of greater numbers of dead cells (trypan blue) after introduction of the higher amount of H_2_O_2_ into the cell cultures. Another explanation could be due to a more subtle effect, based on the interaction between the intracellular signalling system and extracellular reactive oxygen species. In this case, cellular reactivity may have caused changes in cell membranes which resulted in increased exposition of lipid molecules working as a shield against an adverse extracellular environment.

The data could not show the formation of aldehydes and polymerized carbonyl compounds (mentioned by other authors) that occurred during oxidative stress in tissues or cells. Lack of observation of those forms may be connected to the too short interaction time of oxidative species with the cells. Alternatively, these species may be undetectable due to their minor concentration in the investigated material, and optimization of the MS system for particular ions or application of the MRM (multiple reaction monitoring) procedure would allow for successful identification of such modified lipids.

Despite the fact that these results are preliminary, the problem of oxidative stress in the cell culture demands further studies. Our data showed the utility of this type of mass spectrometry imaging for fast analysis of lipids from the cell culture.
